# Seasonal variation of pollen collected by honey bees (*Apis mellifera*) in developed areas across four regions in the United States

**DOI:** 10.1371/journal.pone.0217294

**Published:** 2019-06-12

**Authors:** Pierre Lau, Vaughn Bryant, James D. Ellis, Zachary Y. Huang, Joseph Sullivan, Daniel R. Schmehl, Ana R. Cabrera, Juliana Rangel

**Affiliations:** 1 Department of Entomology, Texas A&M University, Texas, United States of America; 2 Department of Anthropology, Texas A&M University, Texas, United States of America; 3 Entomology and Nematology Department, University of Florida, Gainesville, Florida, United States of America; 4 Department of Entomology, Michigan State University, East Lansing, Michigan, United States of America; 5 Ardea Consulting, Woodland, California, United States of America; 6 Bayer CropScience LP, Crop Science Division, Research Triangle Park, North Carolina, United States of America; Universitat Leipzig, GERMANY

## Abstract

For honey bees (*Apis mellifera*), colony maintenance and growth are highly dependent on worker foragers obtaining sufficient resources from flowering plants year round. Despite the importance of floral diversity for proper bee nutrition, urban development has drastically altered resource availability and diversity for these important pollinators. Therefore, understanding the floral resources foraged by bees in urbanized areas is key to identifying and promoting plants that enhance colony health in those environments. In this study, we identified the pollen foraged by bees in four developed areas of the U.S., and explored whether there were spatial or temporal differences in the types of floral sources of pollen used by honey bees in these landscapes. To do this, pollen was collected every month for up to one year from colonies located in developed (urban and suburban) sites in California, Texas, Florida, and Michigan, except during months of pollen dearth or winter. Homogenized pollen samples were acetolyzed and identified microscopically to the lowest taxonomic level possible. Once identified, each pollen type was classified into a frequency category based on its overall relative abundance. Species richness and diversity indices were also calculated and compared across states and seasons. We identified up to 64 pollen types belonging to 39 plant families in one season (California). Species richness was highest in CA and lowest in TX, and was highest during spring in every state. In particular, “predominant” and “secondary” pollen types belonged to the families Arecaceae, Sapindaceae, Anacardiaceae, Apiaceae, Asteraceae, Brassicaceae, Fabaceae, Fagaceae, Lythraceae, Myrtaceae, Rhamnaceae, Rosaceae, Rutaceae, Saliaceae, and Ulmaceae. This study will help broaden our understanding of honey bee foraging ecology and nutrition in urban environments, and will help promote the use of plants that serve the dual purpose of providing aesthetic value and nutritious forage for honey bee colonies placed in developed landscapes.

## Introduction

Honey bee (*Apis mellifera* L.) workers dedicate most of their foraging phase specializing in the collection of nectar, pollen, propolis, or water [1]. Floral nectar provides the carbohydrates needed for a colony’s energetic needs, while pollen, the main source of protein, provides bees with ten essential amino acids that are critical for brood rearing and queen feeding [2–5]. Pollen is conserved during storage by mixing it with nectar and glandular secretions from workers to create what is known as “bee bread” [6]. Nurse bees consume bee bread to develop their hypopharyngeal glands, which produce a protein-rich jelly that is used to feed developing larvae [7, 8]. Although a colony typically demands more carbohydrates than proteins, pollen can often become a limiting nutritional factor due to low resource availability or quality at certain times of the year [5, 9, 10]. For instance, deficiencies in the availability of particular amino acids can create a bottleneck in brood rearing [5], and without adequate amounts and types of pollen, colonies can quickly deplete their protein reserves, leading to a reduction in brood rearing and even brood cannibalism [11].

The average amount of pollen that a colony with 10,000–15,000 workers needs is estimated at 13.4 to 17.8 kg per year [12, 13]. While having a sufficient amount of pollen is important for colony maintenance, having access to diverse pollen is equally critical to colony nutrition because pollen varies across plant species in the type and amount of amino acids it contains [14–16]. Not surprisingly, polyfloral diets increase worker immunocompetence [17] and overall colony tolerance to pathogens. For example, colonies fed a polyfloral diet exhibit longer worker lifespan by decreasing their susceptibility to the microsporidian gut pathogen *Nosema* spp. [18]. While a few studies suggest that honey bees tend to forage pollen based on the proximity to available floral resources [19–21], others suggest that foragers are capable of displaying pollen preferences based on their colony’s nutritional requirements [22–24]. Nevertheless, it is clear that having access to a consistent flow of pollen from diverse floral sources is beneficial to honey bees.

The recent surge in public awareness regarding the role of honey bee pollination in agriculture has led to an increase in the number of small-scale beekeepers across the U.S., particularly in developed urban and suburban areas [25, 26]. While large-scale commercial beekeeping operations (i.e., those that manage 500 or more colonies) still provide the majority of pollination services to agro-ecosystems, backyard and sideline beekeepers (i.e., those that manage up to 50 or 500 colonies, respectively) represent almost 99% of the beekeeper population in the country [27]. Urban and suburban environments present a different system for colony management compared to rural or agricultural landscapes, given that taxonomic plant diversity can be affected in various ways depending on the degree of land development. For example, heavily developed urban areas are mainly covered with pavement and buildings, resulting in loss of green spaces and reduced availability of plants [28]. In contrast, moderate levels of urbanization may actually increase diversity through irrigated public parks and private gardens [29, 30]. Regardless of the degree of development, many of the small vegetative patches in developed areas often contain few or no native plants because they have been replaced by non-native ornamental plants, which are selected for their aesthetic value rather than for their benefit to pollinators [31]. Urban environments are dominated by few species of ornamental plants that are either competitively dominant or favored in those settings [32, 33]. Although many parks and private gardens in suburban environments contain native and non-native plant species [34], overall, urban development and habitat fragmentation have drastically altered resource availability and diversity for pollinators [35]. Furthermore, landscape transformation is expected to continue, as urbanization is predicted to expand worldwide [36], which will likely have impacts for pollinators due to changes in local flora [37, 38].

To investigate the floral sources collected by honey bee foragers in any landscape, bee-collected pollen can be analyzed with melissopalynology techniques that are typically used to identify pollen in honey to determine its floral sources of nectar [39–44]. However, pollen foragers do not visit the same plants that nectar foragers from the same colony visit, resulting in differences in the types of plants foraged by a colony depending on its nectar and pollen needs [40, 45]. The few studies that have analyzed the composition of pollen pellets collected from foragers have done so using colonies located in predominantly undeveloped or agricultural landscapes [45–47]. Consequently, we have a limited understanding of the pollen foraging preferences of colonies located in urban and suburban environments. In this study, we identified the major pollen types collected by foragers in urban and suburban environments in California, Texas, Florida, and Michigan during the spring, summer, fall, and (when possible) winter months. We used this information to estimate species richness and diversity indices for each site, and compared those values across states and seasons, expecting to find differences in pollen diversity due to spatial and temporal variation between sites. Moreover, because studies in Europe found that colonies placed in agricultural landscapes collected the highest diversity of pollen in the summer months [48–50], we hypothesized that floral diversity in our survey would be highest in the summer season. This study provides insights regarding the foraging ecology and floral preferences of honey bee colonies throughout the year in urban or suburban environments, and serves as a foundation for future work focusing on honey bee foraging ecology and floral preferences in developed landscapes.

## Materials and methods

### Site selection

Beekeepers who managed colonies located in urban and suburban areas were identified through local and state beekeeping organizations in California (CA; Bay Area and Sacramento), Texas (TX; Austin and College Station), Florida (FL; Orlando and Tampa), and Michigan (MI; Detroit and East Lansing). Each owner granted permission to conduct the study on their site. We had a total of 20 sites in CA, 18 sites in TX, 19 sites in FL, and 15 sites in MI, for a total of 394 samples across all sites (Table 1). Each site had at least two colonies so that a focal colony could be replaced in the event that it was later considered unsuitable for acquiring pollen for use in the study.

**Table 1:** Number of sites sampled per state and season. The total number of colonies sampled in each state and the number of samples analyzed in each season. Seasons were defined using the Northern meteorological calendar.

State	No. of colonies	Spring	Summer	Fall	Winter
CA	20	42	44	38	22
TX	18	30	29	20	0
FL	19	27	38	39	40
MI	15	29	45	13	0

The GPS coordinates for each site were collected and mapped on ArcGIS (Version 9.3 or 10.4). Concentric circles were plotted on the map around each site with radii of 0.8 km, 1.6 km and 4.0 km. Assuming honey bees forage in areas closer to the hive [1], a 0.8 km radius around a site was expected to represent the colony’s primary foraging range, while a 4.0 km radius was expected to encompass close to the maximum area where most of the foraging occurs for a typical colony under most circumstances [1]. To account for regional differences in classifying land cover data, we used the 2011 National Land Cover Database (NLCD) to classify the types of land cover surrounding each site (https://www.mrlc.gov/nlcd2011.php). The NLCD classifies developed areas as those with open space, as well as those with low, medium, and high intensity of development or urbanization [51]. The percentages of land cover for each of these four classes were summed to calculate the total percent of developed land around each site.

Undeveloped landscapes consisted of those covered by aquatic systems, shrublands or grasslands, forests and woodlands, croplands, and riparian or wetland systems. Large bodies of water were categorized as unavailable foraging areas so that coastal areas did not underrepresent the level of development in a given area. Agricultural areas were separated into those covered by pasture and hay, and those with cultivated crops. Aerial images of the areas around each colony provided a visual depiction of each site and helped identify landscape features such as residential areas, golf courses, open water, and undeveloped areas. The aerial images were also used to supplement the NLCD to capture recent land development by overlaying the aerial images over the NLCD-classified features. Areas that could be identified as residential or developed using aerial images were manually re-classified as being developed. We then calculated the percentage of developed area out of the total area surrounding the concentric circles around each site. All sites in each state had to be separated from one another by at least four km.

### Pollen collection

We sampled pollen from CA and FL monthly from July 2014 to June 2015 (12 months), MI from July to September 2014 and March to June 2015 (7 months) and TX from March to October 2015 (8 months). We used the Northern meteorological calendar to categorize the data by season based on the month in which they were collected. Spring sampling was done from 1 March to 31 May, summer sampling was done from 1 June to 31 August, fall sampling was done from 1 September to 30 November, and winter sampling was done from 1 December to 28 February.

Each colony had a pollen trap (Brushy Mountain Bee Farm’s item ND#464 for ten-frame hives and item #509 for eight-frame hives) placed at the hive entrance. Participating beekeepers were instructed to activate the pollen traps up to one week prior to the sampling date to collect sufficient pollen from a variety of floral sources. Pollen was passively collected when foragers passed through the activated trap, causing a subset of the pollen pellets on their corbicula to fall into the collecting tray [52]. We sampled pollen within one week or less of activating the traps to get fresh samples that represented the surrounding foraging area from a hive at that particular time. A minimum of 1 g of pollen was collected for palynological analysis from each sampling site at each time point. In total, we collected 146 samples in CA, 79 samples in TX, 144 samples in FL, and 87 samples in MI. The samples were kept frozen at -20°C and shipped on dry ice to Texas A&M University in College Station, TX, for pollen analysis.

### Pollen analysis

Each pollen sample was processed using standard acetolysis procedures [39, 53–56]. Acetolysis is used to remove the protoplasm of a pollen grain so that its morphological characteristics can be visible under a light microscope for taxonomic identification. Subsets of at least 0.25 g of pollen from each sample were homogenized in a test tube with 10 mL of 95% glacial acetic acid, which was added under a fume hood to remove water in the sample, thus preventing a potentially dangerous exothermic reaction between any water residues and the acetolysis mixture. The samples were vortexed and centrifuged at 1060 x *g* for 2 min [56]. After the glacial acetic acid was decanted, 10 mL of the acetolysis mixture containing a 9:1 ratio of acetic anhydride and sulfuric acid was gradually added to the sample. The mixtures were stirred occasionally and were allowed to react on a heating block at 80°C for 10 min. The samples were then topped off with glacial acetic acid and allowed to cool before being centrifuged again at 1060 x *g* for 2 min [56]. After the supernatant was discarded, the samples went through a series of washes with glacial acetic acid and distilled water, and were then stained using Safranin O (Sigma-Aldrich, MO) and rinsed with 95% ethanol. Safranin O is commonly used in palynology because it stains pollen grains in a way that increases the image contrast for microphotography [56–58]. The samples were transferred to Eppendorf tubes containing glycerin and left open for 24 h to dry the ethanol. Slides were made the following day by evenly spreading a drop of pollen residue across the slide and sealing it with a coverslip using clear nail polish [59].

Pollen grains were identified to the family, genus, or species level using a Nikon E200 light microscope. A Nikon DS-L3 stand-alone microscope camera controller was used to measure the size of the pollen grains and to take digital images of unique pollen types at 200× to 600× magnification. Pollen identification was based on the ornamentation, orientation, size, and surface structure of a grain. The specificity of pollen identification depended on the grain's diagnostic characteristics and the reference collection available at the Palynology Laboratory in the Department of Anthropology at Texas A&M University. Various regional pollen atlases were also used and yet, some pollen types were only identified down to the family level. For example, pollen in the Asteraceae, Liliaceae, Poaceae, Rhamnaceae, Rosaceae, and Ericaceae families is not easily identified to the genus level without Scanning Electron Microscope (SEM) images because of the morphological similarity among plants in each of those families [39]. Most members of the Asteraceae family were separated by spine length and differentiated as high spine (HS) Asteraceae (spines > 2.5 μm long), low spine (LS) Asteraceae (spines < 2.5 μm long), or liguliflorae (LF) Asteraceae in the tribe Lactuceae. Taxa were also classified as tree, shrub, herb, combination, or unknown based on the USDA plant database.

We identified at least 200 pollen grains per sample. This number was recently determined to be sufficient to obtain an accurate representation of the plant taxonomic groups present in a forager-collected pollen pellet [60]. Each identified pollen type was placed into one of four frequency categories, as proposed by Louveaux et al. [61]. The “predominant” pollen category included taxonomic groups that were represented in >45% of the pollen grains counted per sample. The “secondary,” “important minor,” and “minor” category included taxonomic groups that were represented in 16–45%, 3–15% and < 3% of the pollen grains counted per sample, respectively [62].

### Taxonomic diversity

Floral taxonomic diversity in each sample was calculated using the Shannon-Weaver diversity index to characterize taxonomic richness and evenness for each season in each state. The Shannon-Weaver diversity index (*H’*) was calculated using the equation:

$$H^{\prime }=-\sum_{i=1}^{S}p_{i}\ln p_{i}$$

where *p*_*i*_ is the proportion of each pollen type (*i*) in the sample and *ln* is the natural logarithm. A greater *H’* value indicates greater taxonomic diversity [62]. Shannon-Weaver diversity indices were calculated for each individual site for each season in every state. We used this index to compare taxonomic diversity at the national and state scale. We also calculated the Effective Number of Species (ENS) from the Shannon-Weaver diversity index for each site to perform biodiversity comparisons between different plant communities, even though the ENS has a tendency to rely on abundant taxa and minimize the input of relatively rare taxa [63]. Unknown pollen types were accounted for by providing each one with a unique “unknown” identification code.

### Statistical analyses

We tested for normality in the Shannon-Weaver diversity indices and the ENS values using a Shapiro-Wilk test. Since the diversity indices for each season and state did not meet the assumptions for normality, we used the nonparametric Kruskal-Wallis test to identify significant differences among diversity values within each season or within each state. We made multiple comparisons for diversity within seasons and states using the Dunn’s test [64]. Because each site had a minimum of two colonies available for sampling, we collected pollen from either the first or second colony at a site despite the possibility that each colony was foraging from different resources [65]. Therefore, each sample was treated as an independent point for our analysis. All descriptive statistics are reported as the mean ± the standard error of the mean (S.E.M.). We set the level of statistical significance for all tests at α = 0.05 and used JMP Pro 14 (SAS Inc., Cary, NC) for all statistical analyses.

## Results

### Verification of developed land cover

**Fig 1:**
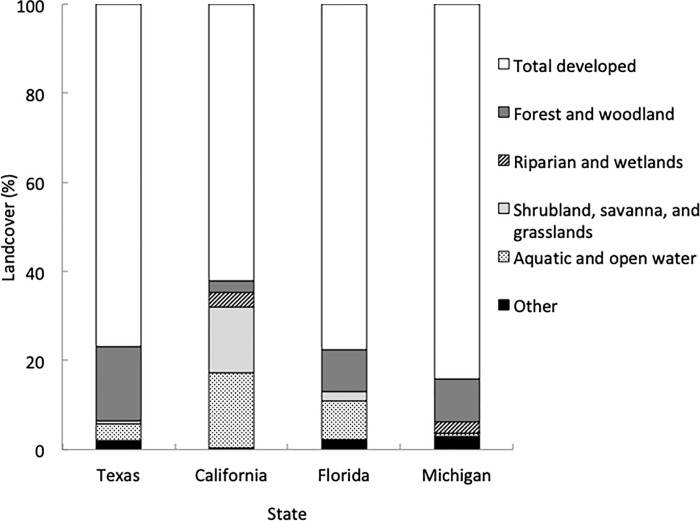
Land cover composition. Overall land cover composition (expressed as percentages) of the environment surrounding honey bee colonies used in each state, using a four-km foraging radius around each colony site. The “other” type of land cover represents cultivated croplands, recently disturbed or modified, pastures or hay, sparse and barren systems, and introduced and semi-natural vegetation.

We used the geographic position of each site and the NLCD information regarding the land cover composition surrounding the site to calculate the percent of land covered by each category outlined previously (i.e., aquatic systems, shrublands or grasslands, forests and woodlands, croplands, and riparian or wetland systems). Using this information, we estimated that across all sites, on average, 81.26% of the land within a four-km radius around each site was developed. The average developed land cover (excluding large bodies of water) surrounding each site was 74.69% in CA, 84.95% in FL, 84.71% in MI, and 80.72% in TX (Fig 1). Assuming a four-km primary foraging radius for foragers in a typical honey bee colony [1], the total surveyed area of potential foraging activity for pollen collection was approximately 3,619 km^2^ across the four states.

### Overall plant species diversity

**Fig 2:**
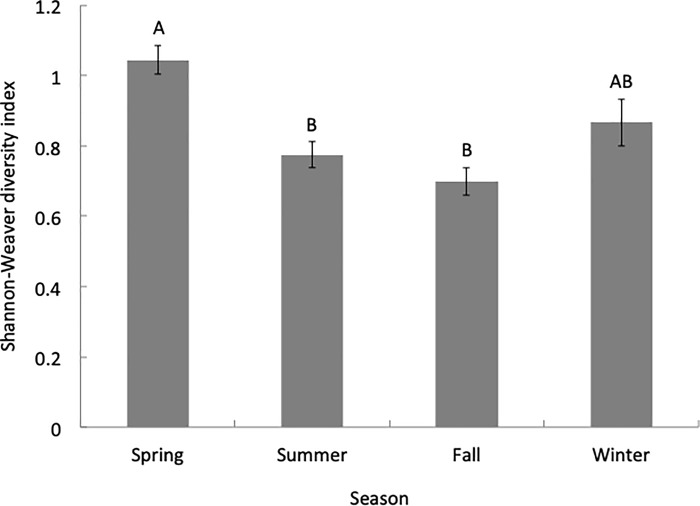
Overall Shannon-Weaver diversity indices by state. Overall Shannon-Weaver diversity index values for all colonies sampled in each state (mean ± S.E.M.).

**Fig 3:**
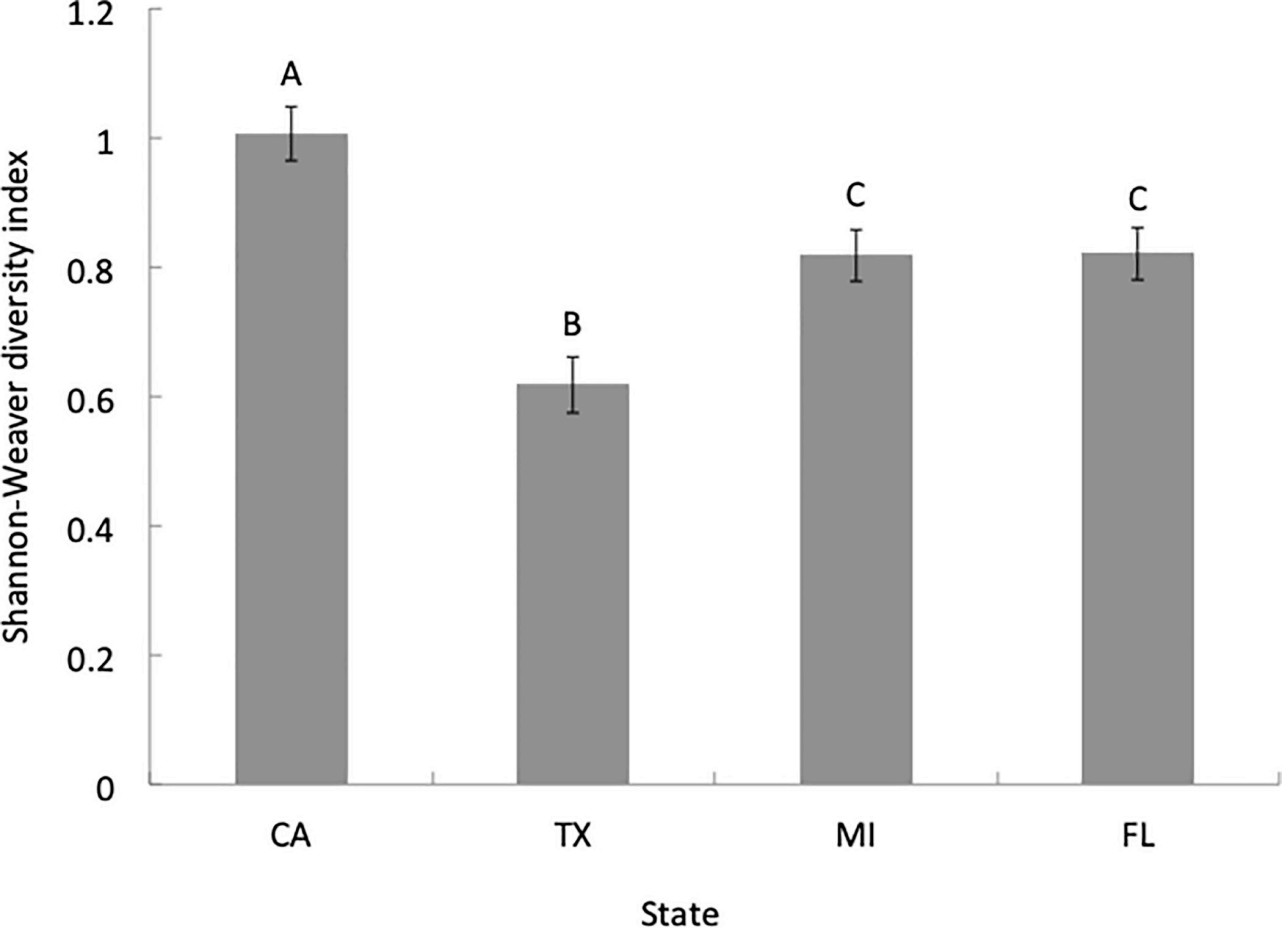
Overall Shannon-Weaver diversity indices by season. Overall Shannon-Weaver diversity index values for all colonies sampled in each season (mean ± S.E.M.).

We found a significant difference in the total overall species diversity across all four states (H = 34.69, *P*<0.001). Post-hoc tests revealed higher overall species diversity in CA and lower overall species diversity in TX compared to other states (Dunn’s all pairs test, *P*<0.05; Fig 2). Nationally, total diversity was also significantly higher in the spring across all locations (H = 33.47, *P*<0.001) compared to other seasons (Dunn’s all pairs test, *P*<0.05; Fig 3).

**Fig 4:**
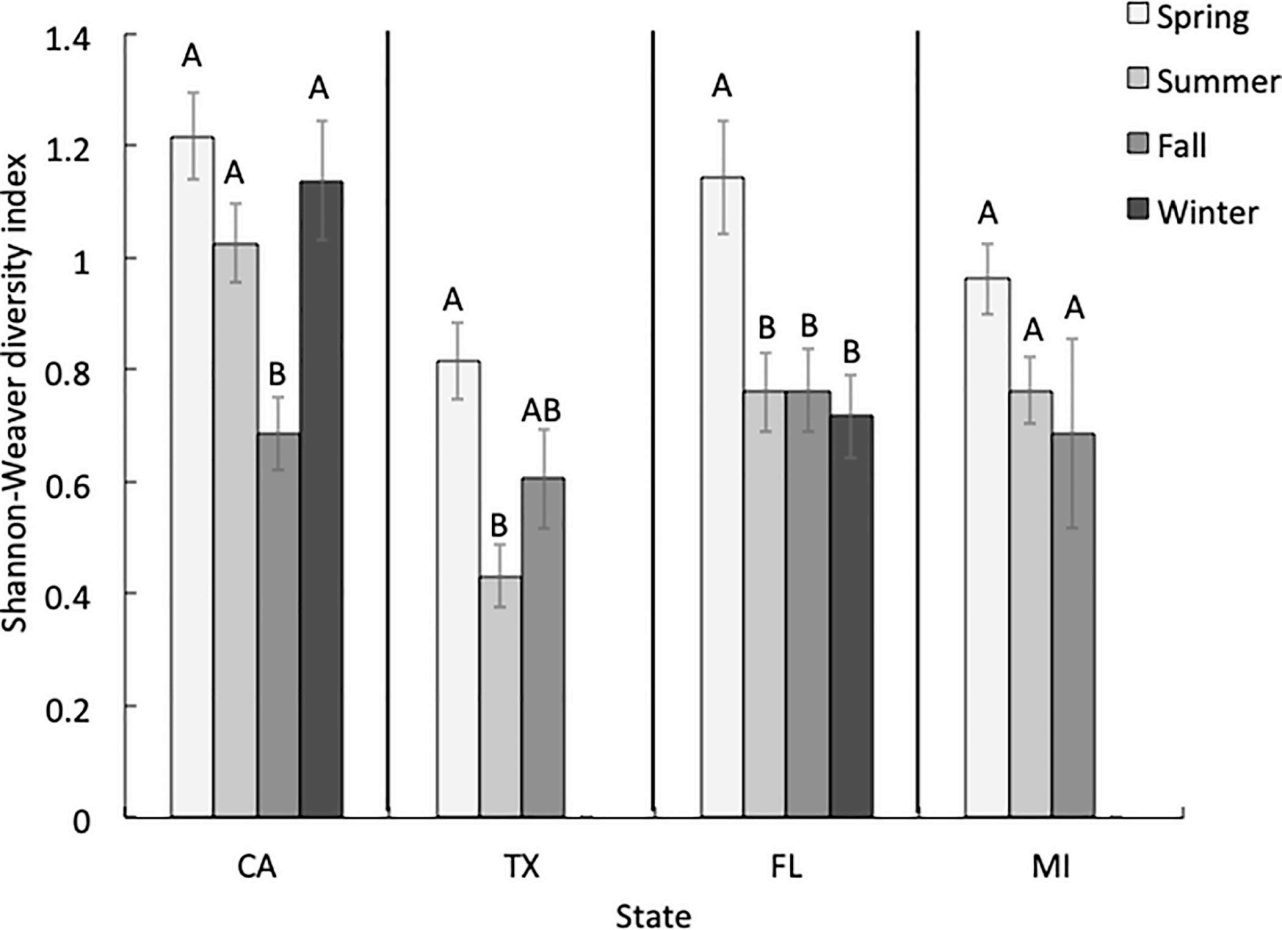
Shannon-Weaver diversity indices by season in each state. Summary of Shannon-Weaver diversity index values (mean ± S.E.M.) for all sites in each state, categorized by season. Different letters above the bars indicate significant differences in Shannon-Weaver diversity indices among seasons within a state.

There were also seasonal differences in species diversity within each state (Fig 4). For example, honey bees in CA collected pollen from significantly fewer (H = 23.67, *P*<0.001) plant species in the fall than in the other seasons (Dunn’s all pairs test, *P*<0.05). In TX, species diversity was significantly higher (H = 12.94, *P* = 0.002) in the spring (H = 12.94, *P* = 0.002) than in the summer (Dunn’s all pairs test, *P*<0.05). Likewise, species diversity in FL was higher (H = 11.66, *P* = 0.009) in the spring than in any other season (Dunn’s all pairs test, *P*<0.05). Although we found a significant effect of seasonal species diversity in MI (H = 6.25, *P* = 0.04), we found no pairwise differences in species diversity among seasons in that state (Dunn’s all pairs test, *P*>0.05).

### Floral diversity in California

**Fig 5:**
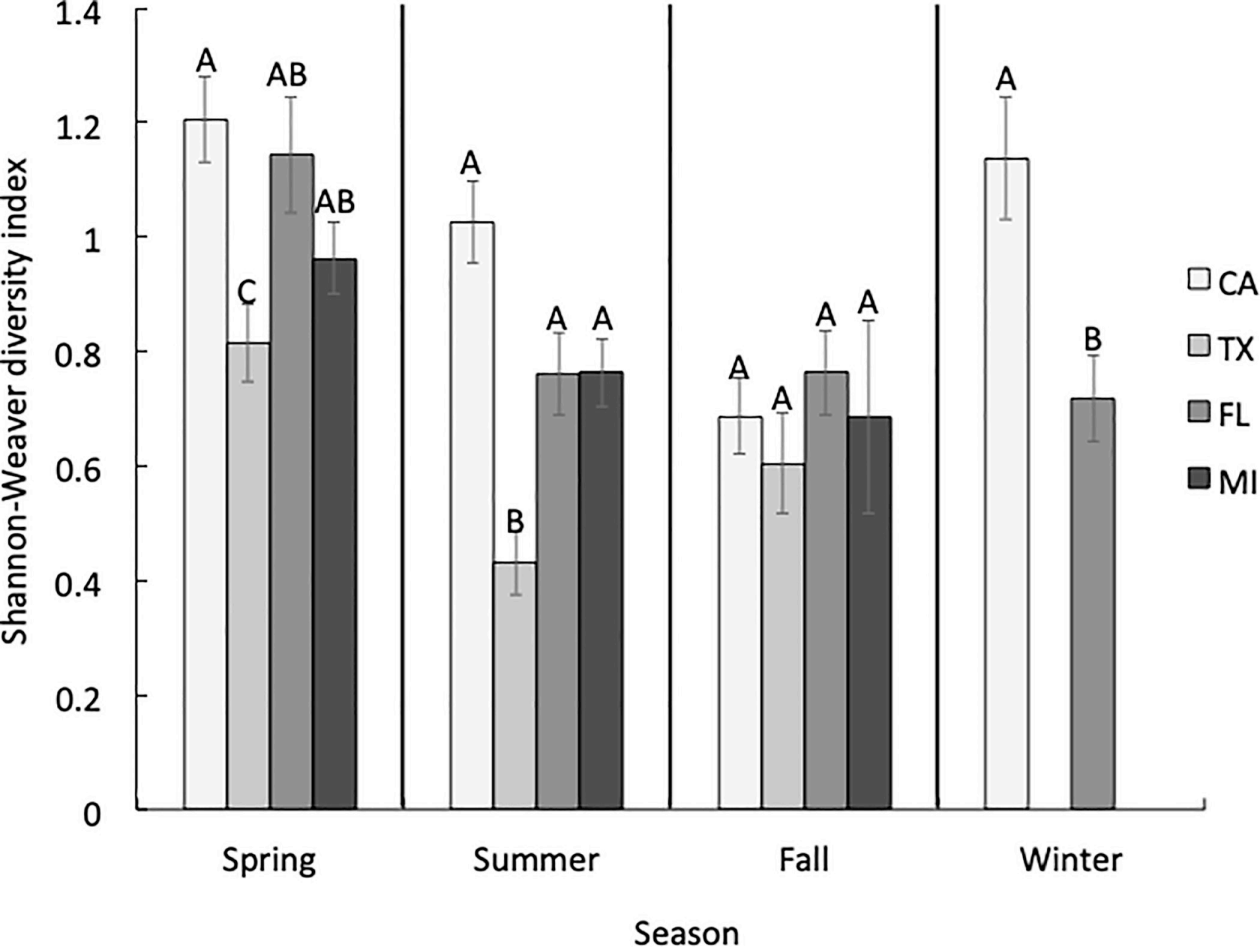
Shannon-Weaver diversity indices by state in each season. Summary of Shannon-Weaver diversity index values (mean ± S.E.M.) for all sites in each season categorized by state. Different letters above the bars indicate significant differences in Shannon-Weaver diversity indices among states within a season.

**Fig 6:**
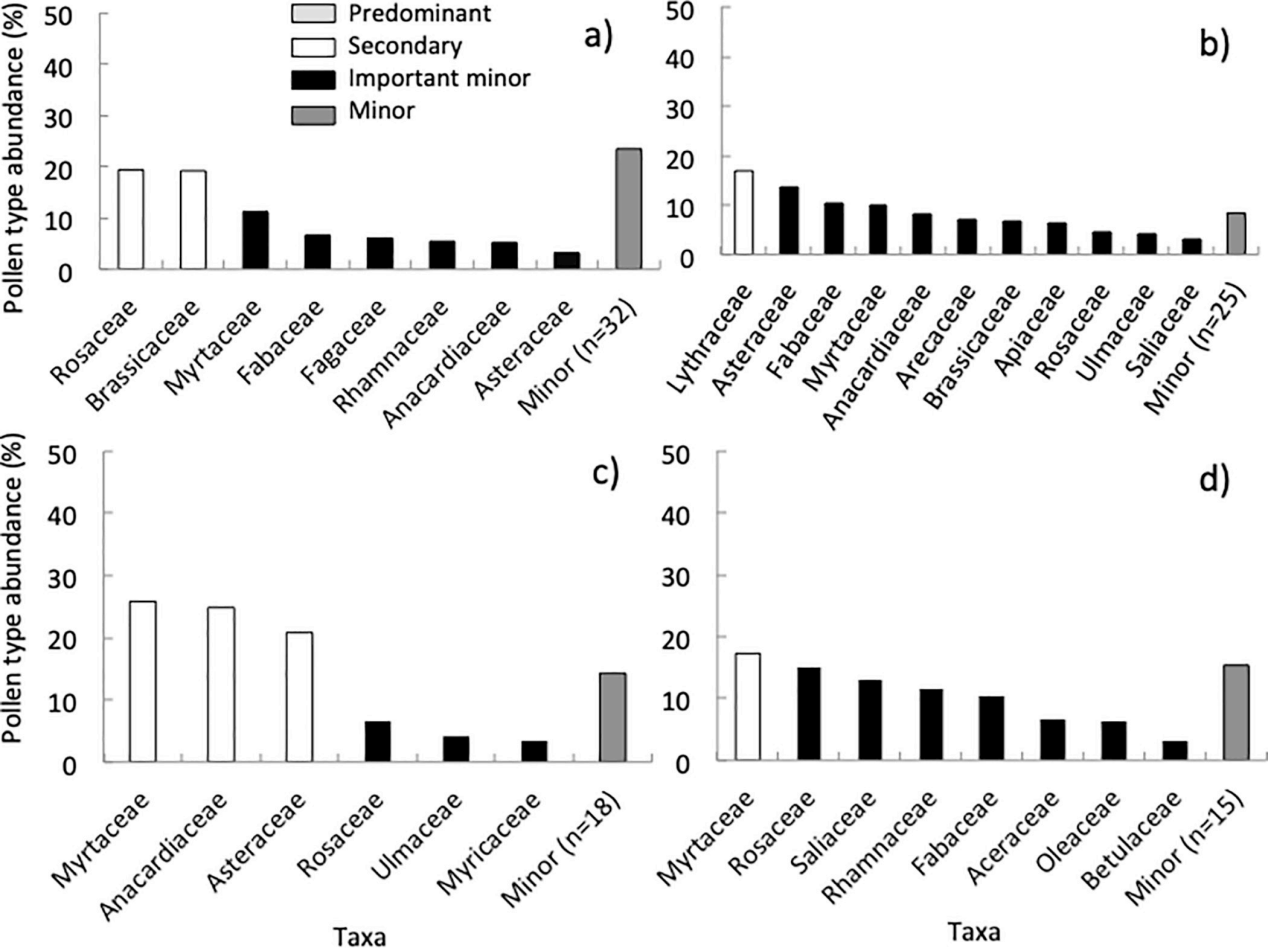
Pollen abundance in California. Summary of the relative abundance of pollen (identified to plant family) collected by honey bee colonies located in urban and suburban environments in CA in the spring (A), summer (B), fall (C), and winter (D). See [61] for an explanation of each abundance category.

In spring, foragers from colonies in CA collected pollen from 65 plant taxa belonging to 39 families (S1 Table, S1 Fig). The average Shannon-Weaver diversity index for each site in the spring was 1.21 and the ENS was 3.75 (Fig 5). None of the pollen collected belonged to the “predominant” category, as proposed by Louveaux et al. [60]. That is, no individual pollen type collected represented >45% of the pollen grains counted in a sample (see “*Site selection*” above for an explanation of each abundance category). The pollen samples consisted of “secondary” and “important minor” plant taxa (Fig 6A). Colonies collected 37.5% of the sampled pollen from herbs and 58.0% from trees and shrubs.

We identified pollen from 48 plant taxa belonging to 34 families collected in the summer (S1 Table, S1 Fig). This included a “secondary” pollen taxon and “important minor” taxonomic groups (Fig 6B). Colonies collected 41.3% of the pollen from herbs, while 58.5% came from trees and shrubs. The Shannon-Weaver diversity index for each site in the summer was 1.03, with an ENS of 3.09 (Fig 5). In the fall, bees collected pollen from 33 taxa belonging to 24 families (S1 Table, S1 Fig). The samples included “secondary” and “important minor” taxa (Fig 6C). Colonies collected 27.1% of the pollen from herbs and 72.4% from trees and shrubs. The average Shannon-Weaver diversity index for each site in the fall was 0.69 and the ENS was 2.14 (Fig 5). Finally, there were 33 taxa belonging to 23 families in the winter (S1 Table, S1 Fig), which belonged to, there was a “secondary” pollen taxon and a few “important minor” taxa (Fig 6D). Colonies collected 18.8% of the pollen from herbs and 69.7% from trees and shrubs. The Shannon-Weaver diversity index for each site was 1.14 and the ENS was 3.46 (Fig 5).

### Floral diversity in Texas

**Fig 7:**
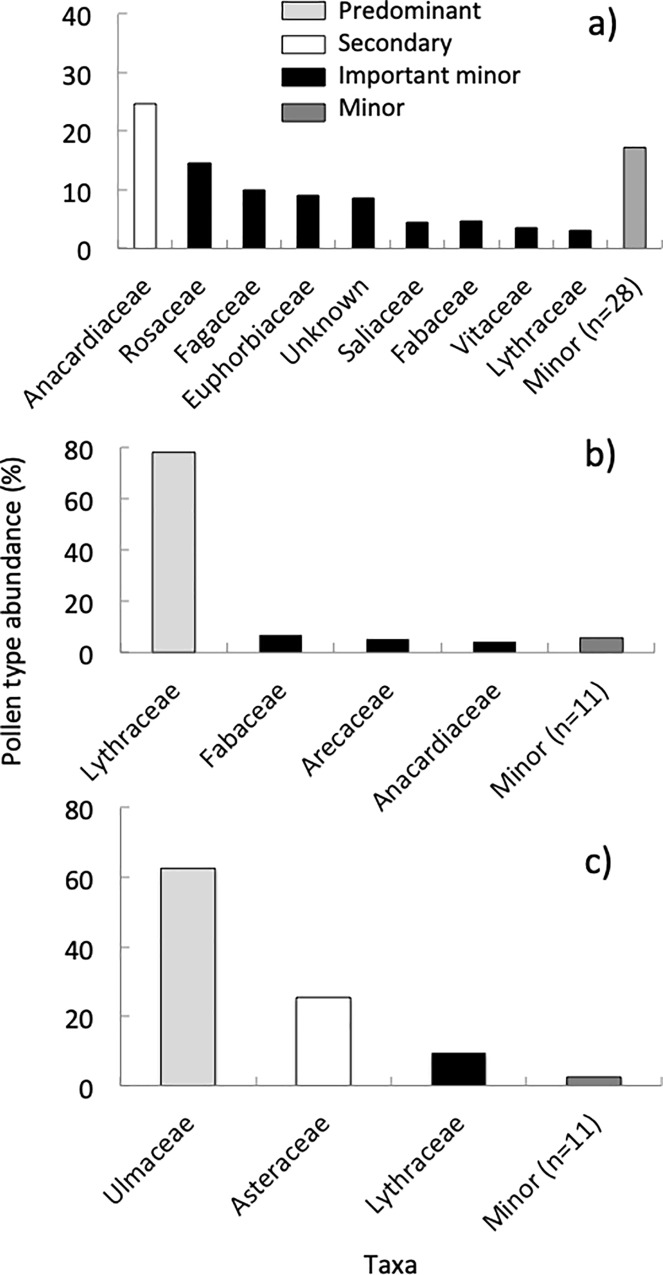
Pollen abundance in Texas. Summary of the relative abundance of pollen (identified to plant family) collected by colonies located in urban and suburban environments in TX during the spring (A), summer (B), and fall (C).

We identified pollen belonging to 49 taxa representing 36 families in the spring (S2 Table, S2 Fig). There was one “secondary” taxon in the Anacardiaceae family, which was also the most abundant plant family found in all the samples. We also found “important minor” plant taxa, as well one important unknown “minor” taxon, which had grains with a tricolporate structure (Fig 7A). Colonies collected 9.5% of the pollen from herbs, while 78.5% came from trees and shrubs. The Shannon-Weaver diversity index for each site in the spring was 0.82, while the ENS was 2.27 (Fig 5).

We found pollen from 20 plant taxa belonging to 15 families in the summer (S2 Table, S2 Fig). We found one “predominant” taxon, as well as “important minor” taxa (Fig 7B). Colonies collected 4.2% of the pollen from herbs and 90.1% from trees and shrubs. The Shannon-Weaver diversity index value for each site in the summer was 0.43 and the ENS was 1.61 (Fig 5). In the fall, we found pollen from 15 plant taxa belonging to 14 families (S2 Table, S2 Fig). These consisted of one “predominant” plant taxon, one “secondary” taxon, and one “important minor” taxon (Fig 7C). Colonies collected 27.6% of the pollen from herbs and 72.4% from trees and shrubs. The Shannon-Weaver diversity for each site in the fall was 0.61 and the ENS was 1.98 (Fig 5). We did not collect any pollen from November to February in Texas.

### Floral diversity in Florida

**Fig 8:**
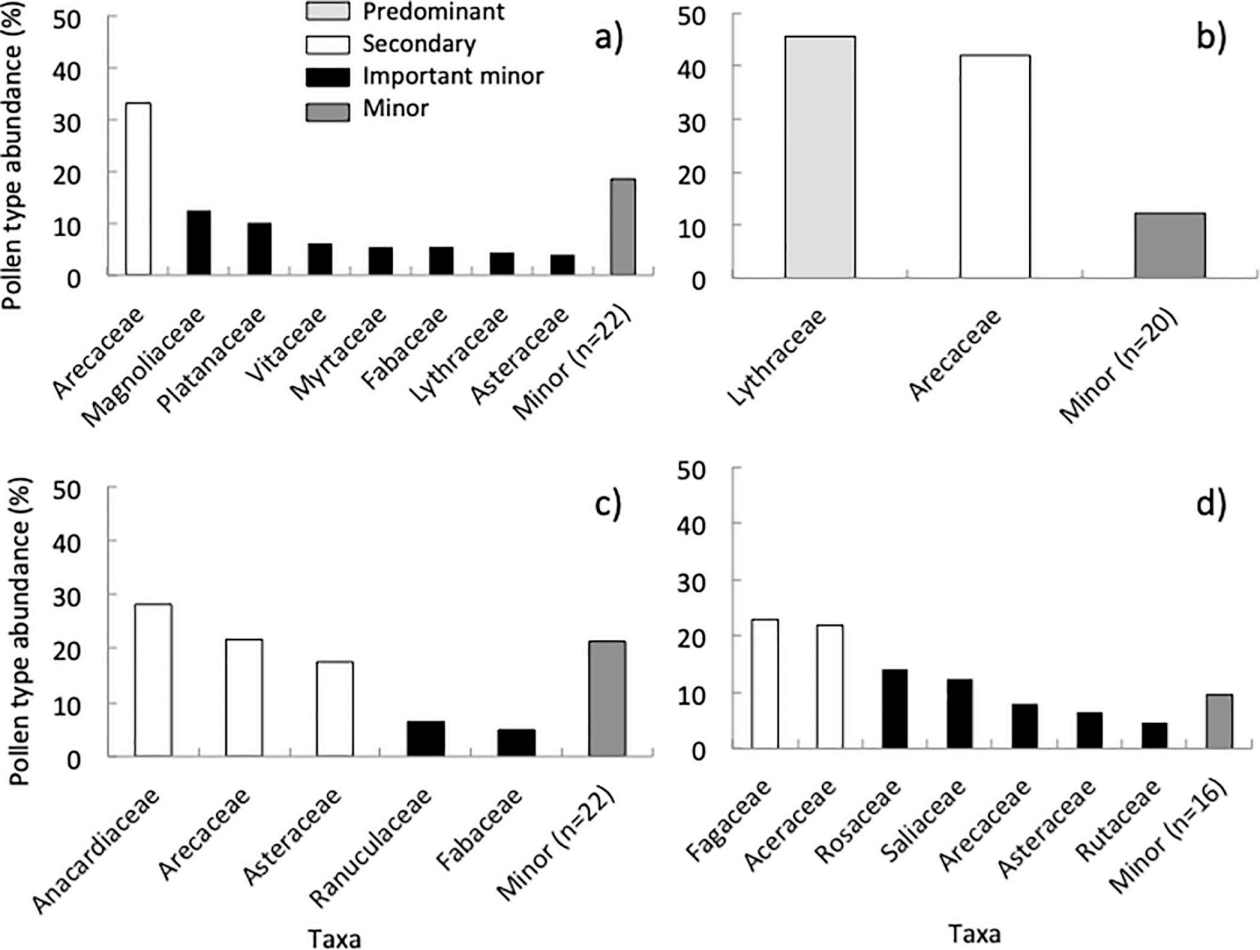
Pollen abundance in Florida. Summary of the relative abundance of pollen (identified to plant family) collected by colonies located in urban and suburban environments in FL during the spring (A), summer (B), fall (C), and winter (D).

We identified 36 plant taxa belonging to 30 families in the spring (S3 Table, S3 Fig), consisting of one “secondary” taxon, and a few “important minor” taxa (Fig 8A). Colonies collected 12.4% of the pollen from herbs and 83.3% from trees and shrubs. The average Shannon-Weaver diversity index for each site in the spring was 1.14, while the ENS was 3.57 (Fig 5). Pollen identified in the summer belonged to 29 plant taxa and 22 families (S3 Table, S3 Fig). These consisted of one “predominant” and one “secondary” plant taxon (Fig 8B). Colonies collected 5.0% of the pollen from herbs, while 94.3% came from trees and shrubs. The Shannon-Weaver diversity index for each site in the summer was 0.76 and the ENS was 2.36 (Fig 5).

In the fall, we found pollen from 34 plant taxa representing 27 families (S3 Table, S3 Fig). There were no “predominant” plant taxa represented in the pollen samples, but we did find “secondary” and “important minor” taxa, as well as one “minor” taxon (Fig 8C). Colonies collected 32.3% of the pollen from herbs and 62.4% from trees and shrubs. The Shannon-Weaver diversity index for each site in the fall was 0.76 and the ENS was 2.14 (Fig 5). Finally, there were 31 different plant taxa in 23 families in the winter (S3 Table, S3 Fig), which belonged to “secondary” and “important minor” taxa (Fig 8D). Colonies collected 6.9% of the pollen from herbs, while 90.6% came from trees and shrubs. The Shannon-Weaver diversity index for each site was 0.72 and the ENS was 2.28 (Fig 5).

### Floral diversity in Michigan

**Fig 9:**
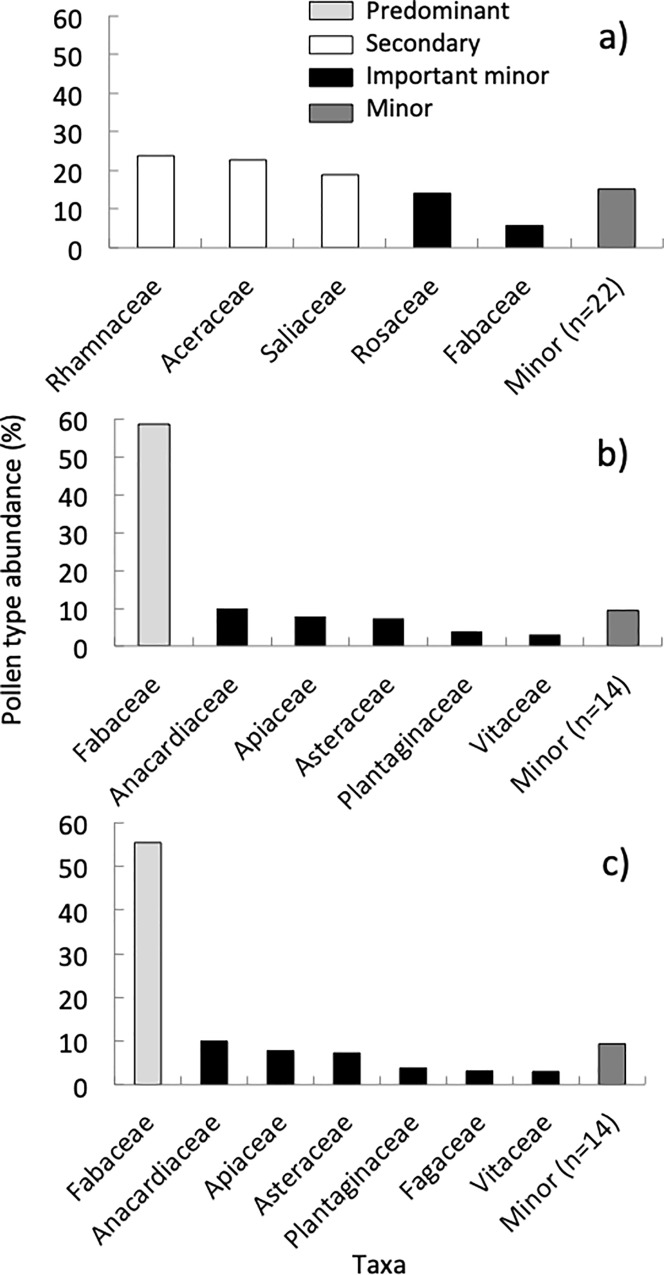
Pollen abundance in Michigan. Summary of the relative abundance of pollen (identified to plant family) collected by colonies located in urban and suburban environments in MI during the spring (A), summer (B), and fall (C).

We identified pollen from 39 taxa and 27 families in the spring (S4 Table, S4 Fig), which belonged to “secondary” and “important minor” plant taxa (Fig 9A). Colonies collected 30.3% of the pollen from herbs and 61.3% from trees and shrubs. The Shannon-Weaver diversity index for each site in the spring was 0.96 and the ENS was 2.61 (Fig 5).

We found 29 plant taxa from 20 families in the summer (S4 Table, S4 Fig), which consisted of “predominant” and “important minor” taxa (Fig 9B). Colonies collected 77.4% of the pollen from herbs and 19.6% from trees and shrubs. The Shannon-Weaver diversity index for each site in the summer was 0.76, while the ENS was 2.32 (Fig 5).

Pollen collected in the fall belonged to 20 taxa from 12 distinct families (S4 Table, S4 Fig). All samples belonging to the Asteraceae family were grouped together because we could not identify them to a lower taxonomic level. Combined, all pollen in the Asteraceae family (55.23%) was considered a “predominant” plant taxon. There were also “important minor” taxa as well as one “important minor” taxon belonging to an unknown taxonomic group with a tricolporate structure, which resembled pollen in the genus *Salix* (Fig 9C). Colonies collected 89.9% of the pollen from herbs, while 3.2% came from trees and shrubs. The Shannon-Weaver diversity index for each site in the fall was 0.69 and the ENS was 2.07 (Fig 5). Pollen was only sampled in September during the fall, and no pollen was collected in the winter.

## Discussion

We conducted a one-year survey of the floral sources of pollen foraged by managed honey bee colonies in urban and suburban environments in four different regions of the U.S. Using standard melissopalynological techniques, we identified pollen pellets collected by foragers in these environments at spatial and temporal scales. Overall, the “predominant” and “secondary” pollen sources collected by foragers across all states originated from plants belonging to various genera in the Fabaceae (legumes), Anacardiaceae (sumac), Lythraceae (loosestrife), Arecaceae (palm), Asteraceae (daisies and asters), Fagaceae (oak), Sapindaceae (soapberry), Rhamnaceae (buckthorn), Salicaceae (willow), Myrtaceae (eucalyptus), Rosaceae (rose), and Brassicaceae (mustard) families (S1 Table, S2 Table). There was also a large representation of pollen from trees and shrubs for colonies in TX, FL, and CA, including those in the families Lythraceae, Arecaceae, Fagaceae, Salicaceae, Myrtaceae, and Rosaceae. These types of trees and shrubs have previously been shown to be important pollen sources for pollinators in urbanized areas [65, 66]. Interestingly, the majority of pollen collected in MI in the summer and fall came from herbaceous plants rather than trees and shrubs, indicating the importance of herbs as honey bee forage in this region of the U.S.

California was the state with the highest number of taxa identified in a given season (n = 64 in the spring). Despite this taxonomic abundance, the relatively high Shannon-Weaver diversity index for that season (1.21) suggests that there was low species evenness at every sampling period in each colony sampled. This indicates that foragers did not collect pollen evenly during a particular sampling period, and instead, likely favored collecting pollen from a few floral resources in each collection. This is consistent with previous work showing that, even in cases when there is a large diversity of plant resources available, honey bees tend to focus their foraging efforts on a few species [46, 67, 68] because the preferred sources are more abundant, or because they provide specific nutrients that colonies need at a particular time. Furthermore, honey bees are known to survey their surrounding area and collectively forage from a few species of plants at a given time until the resource is near exhaustion [1, 45, 49, 67–70].

The range in the observed Effective Number of Species (ENS) across all states and seasons ranged from 1.61 plant taxa in MI during the summer, where pollen in the family Fabaceae was the most “predominant” type, to 3.75 plant taxa in CA during the spring, where most of the pollen collected came from the families Rosaceae, Brassicaceae, Myrtaceae, and Fagaceae. Our study found that the highest diversity of plant taxa foraged occurred in the spring. This result was different from studies in Europe, which found that the highest plant diversity around agricultural landscapes occurs in the summer [48–50].These differences can be attributed to the season, differences in landscape characteristics among study regions, the number of samples collected per site, the lowest floral taxonomic level achieved during pollen identification, or the overall foraging activity of workers. In particular, warmer months in Europe do not occur until later in the year compared to the sites in our study. For example, temperatures in Germany range between 8 ˚C and 18 ˚C in May, which is similar to the temperature in the majority of our sites in the late winter through early springs months. The average latitude across those European studies [48–50], was approximately 48.35° N, whereas the average latitudes for our four states in the U.S. was 35.32° N. In comparison, the amount of pollen collected by honey bee colonies in Israel, which is at a similar latitude as the U.S. (around 31.77° N), was highest from early spring to mid-summer [65].

The low plant taxonomic diversity observed during the summer months in MI is likely due to the abundance of plants grouped in the Fabaceae family, especially in the *Trifolium* and *Melilotis* genera. Even though a colony as a whole may be collecting pollen from several sources of plants over a longer period of time, individual workers typically exhibit temporary specialization and floral constancy for a specific pollen source [4], with 52–79% of the pollen that a colony collects in a week belonging to a single plant species [20]. With a maximum pollen collection period of one week, our methods provided only a limited preview of what colonies are collecting at a given time, as bees could have returned with pollen from completely different plants in the days or weeks before or after sample collection. At times, volunteer beekeepers turned on their traps only a few days before the pollen was sampled. Despite the short window for pollen sampled each month, as well as a colony’s temporary specialization in collecting pollen, we still observed spatial and temporal differences in the diversity of plants foraged by our focal colonies. However, seasonality may have a larger effect on the diversity of pollen collected by foragers compared to landscape level effects, such as the level of urban development in the surrounding landscape of a colony [48].

The spring provided the highest taxonomic richness of pollen in every region, with plants in the families Anacardiaceae, Rosaceae, Rhamnaceae, and Arecaceae representing the majority of pollen collected in TX, CA, MI and FL, respectively. Summer pollen in MI was predominantly represented by genera in the Fabaceae family, particularly plants in the *Trifolium*, *Melilotus*, *Lotus*, *Gleditsia* and *Mimosa* genera, which are common invasive herbs and shrubs found in urban and semi-urban areas [71]. The plant genera found in MI are highly attractive to honey bees, but are considered invasive plants in that state [71]. As the season progressed into the fall, colonies in MI predominantly foraged for pollen in the Asteraceae family, which was also significantly represented as a “secondary” pollen type in CA, TX, and FL. A similar trend of honey bees in the Northern Great Plains transitioning their foraging effort from plants in the Fabaceae family to plants in the Asteraceae family was observed previously by Smart et al. [72].

In CA and FL, where colonies were sampled in the winter, foragers collected pollen mostly from early-blooming trees and shrubs in the Fagaceae, Aceraceae, Myrtaceae, Rosaceae, and Salicaceae families. Only a few plant groups provided pollen reliably throughout the year in all states. For example, plants in the Arecaceae and the Myrtaceae (*Eucalyptus* spp.) families provided a reliable pollen source for colonies the entire year in FL and CA. Plants in the *Eucalyptus* genus and the Arecaceae family are known to be commonly foraged by honey bees and stingless bees in other regions, including Brazil and the West Indies [73, 74]. Pollen from plants in the Arecaceae family tends to contain high levels of protein, essential amino acids and minerals [75]. However, the antioxidant activity of Arecaceae pollen tends to be low when compared to that of other pollen types [76]. Meanwhile, *Eucalyptus* is generally regarded as a nutritionally poor pollen type because it has a low content of lysine (an essential amino acid) and in omega-3 fatty acids, which are positively associated with improved honey bee learning and memory [77, 78].

Crape myrtle, which belongs to the genus *Lagerstroemia* (Lythraceae) was represented as a “predominant” plant type foraged in TX and FL, and was a “secondary” plant type in CA. A deciduous shrub native to India, crape myrtle is a common ornamental plant in urban environments that displays dimorphic pollen with two distinct whorls: one that provides food to pollinators, and one that is used for plant fertilization [79, 80]. Interestingly, although crape myrtle pollen has been previously found in honey from a few states [81], its presence in honey is likely a spillover effect resulting from “cross-contamination” from pollen foragers, given that this plant lacks nectaries and thus, it is not a source of nectar for bees. Crape myrtle has been documented as a pollen source for native and non-native bees in other studies [82–85]. In fact, crape myrtle may be undervalued for its contributions to honey bee nutrition in the late summer months, a period when colonies can experience severe resource dearth [86]. Therefore, the “predominant” presence of crape myrtle pollen we observed in the summer suggests that some of our study colonies relied on the presence of this ornamental plant in urban environments for pollen acquisition [80, 87].

Maintaining a reliable flow of pollen when brood rearing is critical for nutrition [88]. In a heterogeneous environment containing food sources varying in quality and quantity, honey bees adopt different foraging strategies to regulate their nutritional state [89]. For example, honey bees prefer certain essential amino acids and fatty acids over others, as well as certain diets that complement previous nutritionally deficient diets [22–24, 90]. In our study, we qualified the types of plants foraged by bees in urban environments using a method of categorization previously described [61], and addressed the spatial and temporal differences in pollen diversity. However, since we did not quantify the relative amount of pollen brought in from each floral source, our results can neither infer a colony’s nutritional state nor determine whether or not colonies in urban and suburban environments are pollen limited. Avni et al. [13] found spatial and temporal differences in pollen quantity and content by measuring colony pollen intake and quantifying the macronutrients therein for an entire year [13]. Despite these differences, the authors did not find any nutritional effects of pollen quantity and quality on overall colony growth on a yearlong basis. As generalist foragers, honey bees collect resources from a wide range of plants and can compensate for nutritional deficiencies by collecting complimentary diets [23]. Future studies to address this question further should incorporate the use of The Geometric Framework, which explores how organisms (including individuals and colony-living “superorganisms”) balance their nutrient intake on a multidimensional nutrient space [91–93].

Our main objective in this study was to identify the “predominant,” “secondary,” and “important minor” plant taxonomic groups from which honey bees collect pollen in urban and suburban areas. One limitation was that not all pollen types were identified to the species level, in part because the traditional light microscopy method we used to identify pollen based on grain morphology is a time-consuming process that requires a high level of expertise. Depending on the microscope and time constraints of a desired study, Scanning Electron Microscopy (SEM) could be used in conjunction with light microscopy to visualize certain morphological features that are unclear with light microscopy alone, providing better taxonomic resolution to the genus or species level [39, 94]. Newer technologies such as DNA meta-barcoding, which screens pollen samples for specific plant genome sequences, are currently being explored for pollen identification [68, 95–98]. However, this method is still in development, as it does not adequately provide the relative abundance of each pollen type, and it can provide “false positives” in the plant identification results [98, 99]. A combined use of microscopy and molecular techniques would likely provide the most thorough and reliable information when performing a palynological analysis of floral origins and pollen grain abundance in a given sample. Although we only sampled pollen in a limited time frame within each month, our results are representative of the plant sources of pollen preferred in developed environments across different seasons and geographic regions. With the ever-changing composition of landscapes, our results can help us better understand honey bee nutritional ecology in urban and suburban environments, and can aid in promoting the use of plants that provide appropriate pollen resources to honey bees in developed areas. In addition, the data generated in this study could be used by public and private urban gardeners to aid them in the selection of pesticides used on ornamental and landscaped plants, especially in terms of the timing and application rates of products used around homes and gardens. For example, pesticide treatment regimens should be planned to avoid chemical application when bees are primarily foraging from a given plant for pollen. Similar studies in other regions of the country are encouraged to help us better understand, and potentially improve, the foraging conditions available to honey bees in urban environments throughout the year.

## Supporting information

S1 Table Taxonomic identification of pollen collected in California. The family, genus, frequency class, grain count (number of pollen grains counted), relative abundance (as described by Louveaux et al. [58]) and general type of the plant sources of pollen identified in California for each season. Predominant, secondary, important minor, and minor pollen types are defined as those taxonomic groups detected in over 45%, 16–45%, 3–16%, or less than 3%, respectively, of the 200 pollen grains analyzed per sample, as described previously [58].
(XLSX)

S2 Table Taxonomic identification of pollen collected in Texas. The family, genus, frequency class, grain count (number of pollen grains counted), relative abundance (as described by Louveaux et al. [58]) and general type of the plant sources of pollen identified in Texas for each season. Predominant, secondary, important minor, and minor pollen types are defined as those taxonomic groups detected in over 45%, 16–45%, 3–16%, or less than 3%, respectively, of the 200 pollen grains analyzed per sample, as described previously [58].
(XLSX)

S3 Table Taxonomic identification of pollen collected in Florida. The family, genus, frequency class, grain count (number of pollen grains counted), relative abundance (as described by Louveaux et al. [58]) and general type of the plant sources of pollen identified in Florida for each season. Predominant, secondary, important minor, and minor pollen types are defined as those taxonomic groups detected in over 45%, 16–45%, 3–16%, or less than 3%, respectively, of the 200 pollen grains analyzed per sample, as described previously [58].
(XLSX)

S4 Table Taxonomic identification of pollen collected in Michigan. The family, genus, frequency class, grain count (number of pollen grains counted), relative abundance (as described by Louveaux et al. [58]) and general type of the plant sources of pollen identified in Michigan for each season. Predominant, secondary, important minor, and minor pollen types are defined as those taxonomic groups detected in over 45%, 16–45%, 3–16%, or less than 3%, respectively, of the 200 pollen grains analyzed per sample, as described previously [58].
(XLSX)

S5 Table Diversity indices data. Raw data for the diversity indices calculated at each sampling point.
(XLSX)

S1 Fig Photomicrographs of CA predominant (P), secondary (S), and important minor (IM) pollen types. 1) Rosaceae type, 2) *Brassica*, 3) Myrtaceae type, 4) *Quercus*, 5) Rhamnaceae type, 6) *Rhus*, 7) *Vicea*, 8) Medicago, 9) Asteraceae- Lactuceae, 10) *Lagerstroemia indica*, 11) *Centaurea*, 12) *Trifolium/Melilotus*, 13) *Lotus*, 14) *Eucalyptus*, 15) *Rhus*, 16) Arecaceae type, 17) *Brassica*, 18) Apiaceae type, 19) *Prunus*, 20) *Ulmus*, 21) *Salix*, 22) *Eucalyptus*, 23) Anacardiaceae type, 24) HS Asteraceae, 25) Rosaceae type, 26) *Ulmus*, 27) *Myrica*, 28) *Eucalyptus*, 29) *Prunus*, 30) *Salix*, 31) Rhamnaceae type, 32) *Acacia*, 33) *Acer*, 34) *Fraxinus*, 35) *Alnus*. The white scale within each box represents 25 μm.
(TIF)

S2 Fig Photomicrographs of TX predominant (P), secondary (S), and important minor (IM) pollen types. 1) *Rhus*, 2) *Crataegus*, 3) *Quercus*, 4) *Triadaca sebifera*, 5) Unknown tricolpate type, 6) *Populus*, 7) *Trifolium*/*Melilotus*, 8) *Vitis*, 9) *Lagerstroemia indica* 10) *Lagerstroemia indica*, 11) *Prosopis*, 12) Areaceae type, 13) *Rhus*, 14) *Lagerstroemia indica*, 15) HS Asteraceae, 16) *Ulmus*. The white scale within each box represents 25 μm.
(TIF)

S3 Fig Photomicrographs of FL predominant (P), secondary (S), and important minor (IM) pollen types. 1) Arecaceae type, 2) *Magnolia grandiflora*, 3) *Platanus*, 4) *Vitis*, 5) *Eucalyptus*, 6) *Mimosa pudica*, 7) *Lagerstroemia indica*, 8) HS Asteraceae type, 9) *Lagerstroemia indica*, 10) Arecaceae type, 11) *Schinus*, 12) *Cocos*, 13) HS Asteraceae, 14) Ranunculaceae type, 15) *Casuarina*, 16) *Baptisia*, 17) *Quercus*, 18) *Acer*, 19) Rosaceae type, 20) *Salix*, 21) Arecaceae type, 22) HS Asteraceae type, 23) *Citrus*. The white scale within each box represents 25 μm.
(TIF)

S4 Fig Photomicrographs of MI predominant (P), secondary (S), and important minor (IM) pollen types. 1) Rhamnaceae type, 2) *Acer*, 3) *Salix*, 4) *Prunus* 5) *Robinia*, 6) *Trifolium/Melilotus* type 1, 7) *Trifolium*/*Melilotus type 2*, 8) *Castanea*, 9) *Rhus*, 10) Apiaceae type, 11) *Plantago*, 12) *Parthenocissus*, 13) HS Asteraceae, 14) HS Asteraceae - Lactuceae tribe, 15) *Artemisia*, 16) Ranunculaceae type, 17) *Trifolium*, 18) Unknown tricolpate type, 19) Poaceae type. The white within each box scale represents 25 μm.
(TIF)
