# Supplementary figures and images for: Seasonal variation of pollen collected by honey bees (Apis mellifera) in developed areas across four regions in the United States
Source: PLoS One. 2019 Jun 12;14(6):e0217294. doi: 10.1371/journal.pone.0217294 (PMC6561680; doi:10.1371/journal.pone.0217294)

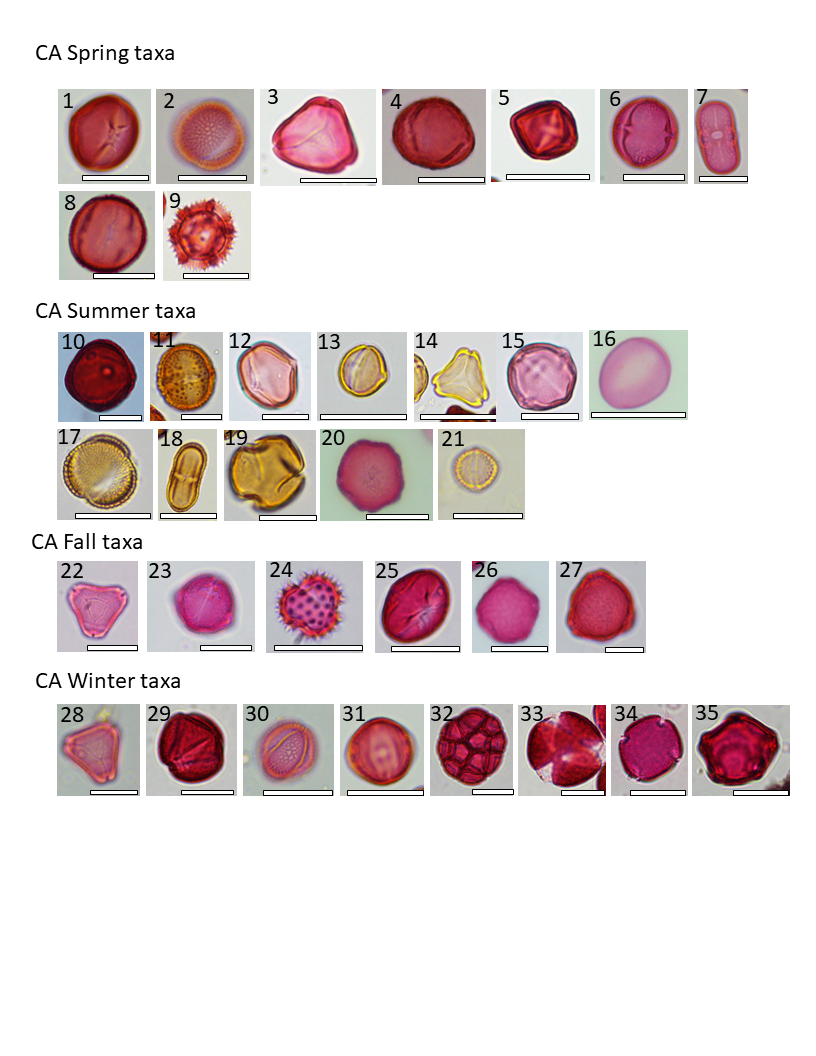

Supplement: S1 Fig — 1) Rosaceae type, 2) Brassica, 3) Myrtaceae type, 4) Quercus, 5) Rhamnaceae type, 6) Rhus, 7) Vicea, 8) Medicago, 9) Asteraceae- Lactuceae, 10) Lagerstroemia indica, 11) Centaurea, 12) Trifolium/Melilotus, 13) Lotus, 14) Eucalyptus, 15) Rhus, 16) Arecaceae type, 17) Brassica, 18) Apiaceae type, 19) Prunus, 20) Ulmus, 21) Salix, 22) Eucalyptus, 23) Anacardiaceae type, 24) HS Asteraceae, 25) Rosaceae type, 26) Ulmus, 27) Myrica, 28) Eucalyptus, 29) Prunus, 30) Salix, 31) Rhamnaceae type, 32) Acacia, 33) Acer, 34) Fraxinus, 35) Alnus. The white scale within each box represents 25 μm. (TIF) [file pone.0217294.s006.tif]

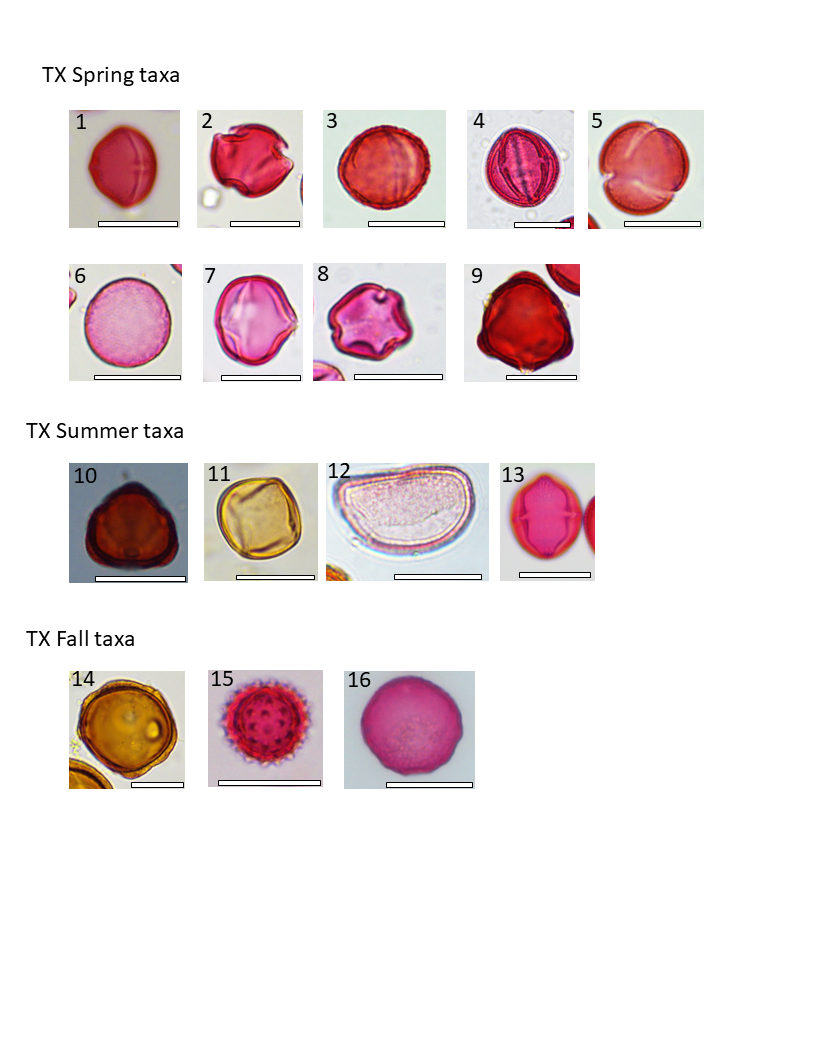

Supplement: S2 Fig — 1) Rhus, 2) Crataegus, 3) Quercus, 4) Triadaca sebifera, 5) Unknown tricolpate type, 6) Populus, 7) Trifolium/Melilotus, 8) Vitis, 9) Lagerstroemia indica 10) Lagerstroemia indica, 11) Prosopis, 12) Areaceae type, 13) Rhus, 14) Lagerstroemia indica, 15) HS Asteraceae, 16) Ulmus. The white scale within each box represents 25 μm. (TIF) [file pone.0217294.s007.tif]

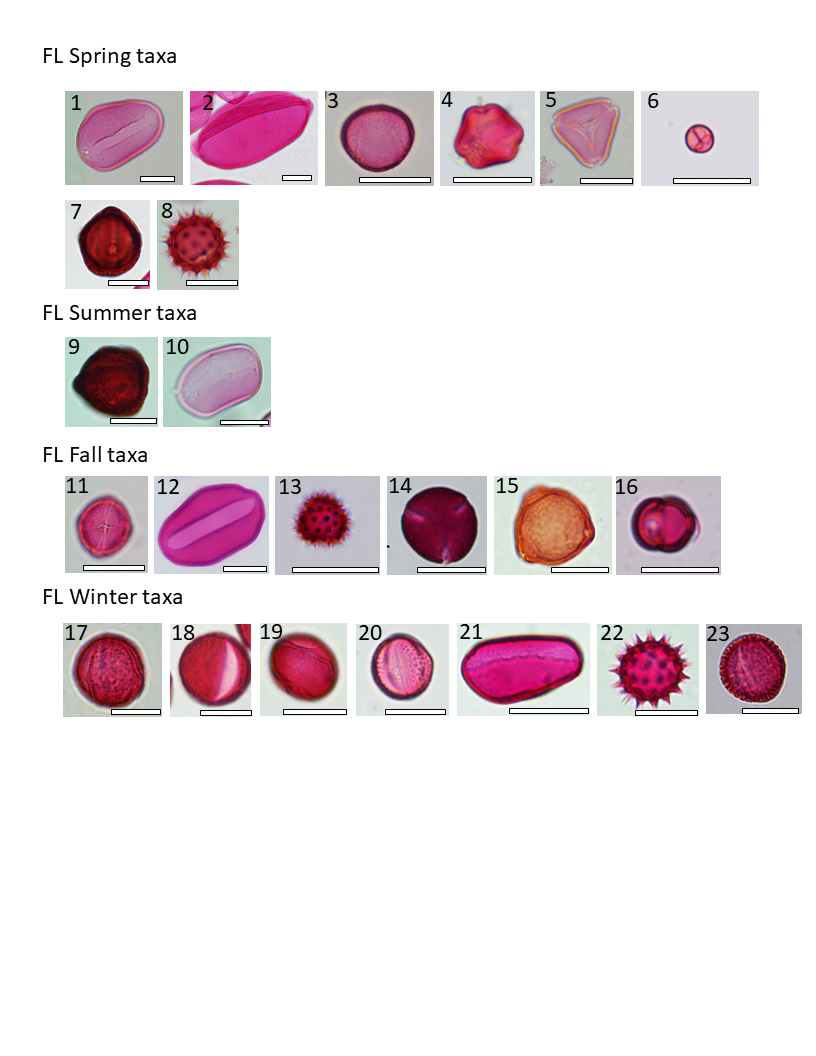

Supplement: S3 Fig — 1) Arecaceae type, 2) Magnolia grandiflora, 3) Platanus, 4) Vitis, 5) Eucalyptus, 6) Mimosa pudica, 7) Lagerstroemia indica, 8) HS Asteraceae type, 9) Lagerstroemia indica, 10) Arecaceae type, 11) Schinus, 12) Cocos, 13) HS Asteraceae, 14) Ranunculaceae type, 15) Casuarina, 16) Baptisia, 17) Quercus, 18) Acer, 19) Rosaceae type, 20) Salix, 21) Arecaceae type, 22) HS Asteraceae type, 23) Citrus. The white scale within each box represents 25 μm. (TIF) [file pone.0217294.s008.tif]

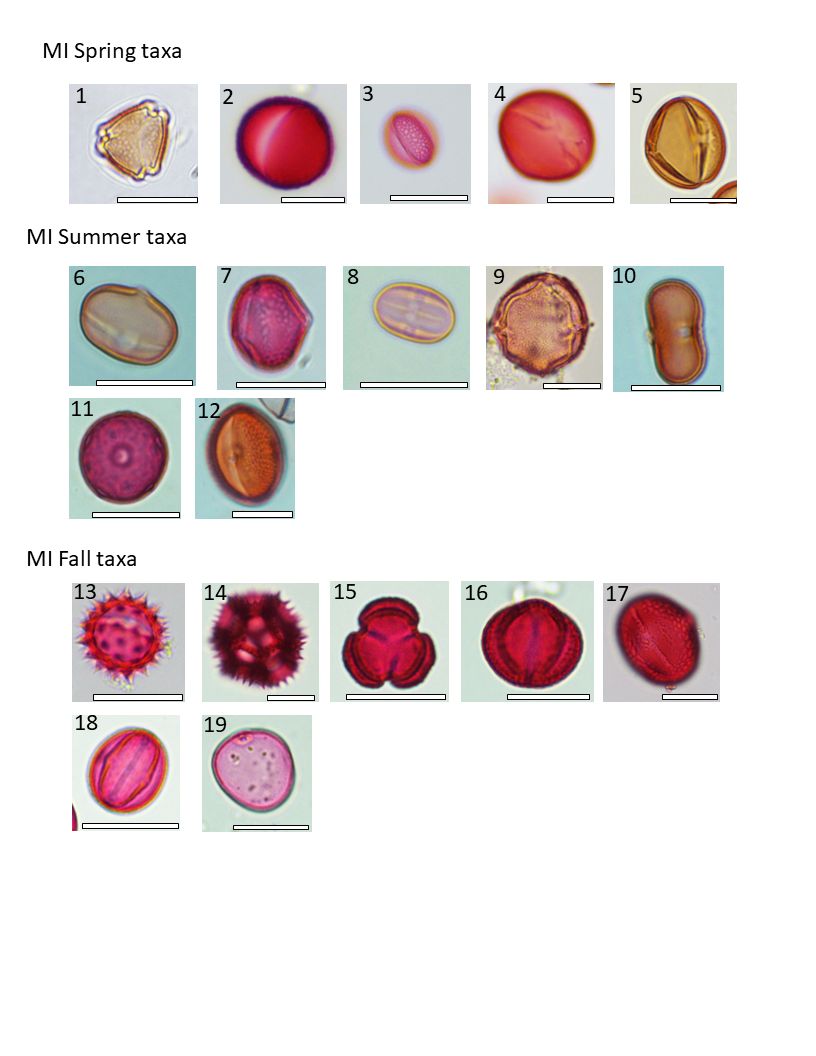

Supplement: S4 Fig — 1) Rhamnaceae type, 2) Acer, 3) Salix, 4) Prunus 5) Robinia, 6) Trifolium/Melilotus type 1, 7) Trifolium/Melilotus type 2, 8) Castanea, 9) Rhus, 10) Apiaceae type, 11) Plantago, 12) Parthenocissus, 13) HS Asteraceae, 14) HS Asteraceae - Lactuceae tribe, 15) Artemisia, 16) Ranunculaceae type, 17) Trifolium, 18) Unknown tricolpate type, 19) Poaceae type. The white within each box scale represents 25 μm. (TIF) [file pone.0217294.s009.tif]
